# Psychometric validation of the self-compassion scale and the link of self-compassion to managerial flourishing in South Africa

**DOI:** 10.1186/s40359-025-02922-7

**Published:** 2025-06-03

**Authors:** Mari Ford, Sebastiaan Rothmann, Llewellyn van Zyl

**Affiliations:** 1 Department of Applied Management, Administration & Ethical Leadership, University of Fort Hare, East London, South Africa; 2 Optentia Research Unit, North‒West University, Vanderbijlpark, Gauteng South Africa

**Keywords:** Psychometric properties, Self-compassion, Well-being, Flourishing, Managers, Organizations, South Africa

## Abstract

**Background:**

Self-compassion is a critical personal resource that can assist managers in addressing the demands of their jobs and flourishing. This study investigated the factor validity, reliability, and measurement invariance of Neff’s Self-Compassion Scale across genders and the association between self-compassion and managers’ workplace flourishing using a person-centered approach.

**Method:**

Managers (*N* = 390) registered for postgraduate qualifications at business schools across South Africa participated in a cross-sectional survey. Participants completed an online questionnaire, which included the Self-Compassion Scale and the Flourishing-at-Work Scale–Short Form.

**Results:**

The results supported a bifactor exploratory structural equation model with one global factor and six well-defined subfactors of self-compassion. The measurement invariance of the scale was established across genders. Latent profile analysis identified four flourishing profiles: languishers, moderate languishers, moderate flourishers, and flourishers. Significant differences were found between the self-compassion of managers in these profiles. Flourishers reported the highest levels, while languishers reported the lowest levels of self-compassion.

**Conclusions:**

Neff’s self-compassion scale is valid and reliable and should be retained in its original form. Self-compassion is closely linked with managers’ flourishing and should be included in all management development programs to improve well-being. This may be an essential way to promote flourishing in organizations.

## Background

Managers in organizations experience pressure from many different sources. On the one hand, there are pressures such as creating shareholder value, delivering against performance and efficiency targets, and dealing with the impact of fast-changing business environments [1–3]. On the other hand, there are pressures such as operating in a post-COVID world, dealing with high levels of employee anxiety and disengagement, and increasing calls for ethical, authentic, and transparent leadership [4, 5]. The impact of these pressures on business managers can mean increased stress, burnout, reduced job satisfaction, and an inability to perform at their best [6]. In short, managers may start languishing instead of flourishing, negatively affecting their colleagues, employees, and organizations.

One concept that has consistently been associated with a decrease in stress, anxiety, and depression is self-compassion. Self-compassion has been linked to decreased negative and increased positive affect, more ethical decision-making, and closer social relationships [7, 8]. Self-compassion has been closely related to all aspects of well-being and could be an essential personal resource, helping managers to deal more effectively with the pressures of their jobs, make better decisions, and be more successful [9–11].

Research on self-compassion has focused on emotional well-being, showing significantly positive correlations with positive affect, happiness, optimism, and life satisfaction [12, 13] and significantly negative correlations with depression and personal distress [14–16]. In terms of psychological well-being, self-compassion relates to greater mental well-being [17], higher quality of life [18], greater wisdom and curiosity [12], and increased health-promoting behaviors [19]. Self-compassion has also been linked to social well-being by improving social relationships and interactions [8, 17, 20].

Self-compassion is described as both a trait and a state. It can be an attitude or a characteristic that informs the way people behave toward themselves at any given moment, but it can also be a state that can be intentionally practised and developed over time, as several intervention studies have shown. This makes it a valuable resource for personal growth and development [11, 21, 22].

Several measures of self-compassion exist within the literature, but the Self-Compassion Scale (SCS) designed by Neff [23] is by far the most popular and widely used for research in organizational contexts. Despite its importance, debates still exist about the factor structure of the SCS, and more research is essential to test its validity and reliability in different contexts. Similarly, more research is needed to understand the role of self-compassion in the flourishing of managers [10], which is especially true in non-Western, Educated, Industrialized, Rich, and Democratic (WEIRD) contexts such as South Africa. The purpose of this study was twofold: first, to examine the psychometric properties of the SCS and, second, to explore the relationship between self-compassion and the flourishing of managers using a person-centered approach.

### Measurement of self-compassion

Self-compassion is often described as compassion turned inwards [23]. Self-compassionate individuals acknowledge their suffering and then, in a kind and caring way, take steps to help themselves and alleviate their pain in the same way that they might help a loved one or family member [22]. The concept stems from Buddhist philosophy but was not initially viewed as different from a general sense of compassion until 20 years ago when Neff’s pioneering work operationalized it separately [23]. To align the concept of self-compassion with its Buddhist meaning, the original construct included a general self-compassion factor with six subscales: three positive and three negative subscales [23]. These subscales relate to three main underlying components, each describing either a positive or a negative end of a continuum [24]. The first component, *self-kindness*, refers to individuals’ attitudes of kindness, friendliness, and supportiveness toward themselves [23]. This is seen in relation to *self-judgment*, in which people judge themselves harshly for mistakes they might have made or for the behavior they perceive to be bad [22]. The second component relates to *common humanity*, which involves recognizing that we share experiences, emotions, and difficulties as humans. This aspect cultivates an acceptance that failure, making mistakes, and trying our best are part of the shared human experience. At the opposite end of this continuum is *isolation*, in which individuals feel isolated by their mistakes or misfortunes and believe that they are the only people to suffer in this way [25]. The third component, *mindfulness*, involves people being aware of their moment-by-moment experience of suffering by tuning into their suffering and trying to understand it more compassionately and clearly. At the opposite of the mindfulness continuum is *overidentification*, in which individuals develop an obsessive way of thinking about their difficulties, repeating the same negative thoughts in their minds [23, 26].

The SCS has been validated and tested in multiple contexts, in different countries, and with many different groups of people [27–29]. Although this usually implies consistency and uniformity among researchers, there is still considerable debate about the construct of self-compassion and how best to analyze and measure it. Specifically, the construct, as Neff [23] operationalized it, with a general self-compassion factor, plus six subfactors that operate along three continuums, has not been universally supported.

### Factorial validity of the SCS

While many studies have supported the six-factor first-order model, several have shown no support for the higher-order general self-compassion factor [28, 30]. Furthermore, some studies have found a better model fit using two distinct factors: positive factors (called compassionate self-response or self-compassion) and negative factors (called uncompassionate self-response or self-coldness) [31–34]. These findings have led researchers to question whether the scale should include positive and negative subscales and whether the negative items might overinflate the relationship between self-compassion and psychopathology [35, 36]. This argument questions the structure and validity of the SCS and is, therefore, a crucial issue about which Neff has written on many occasions [24, 26, 37].

Neff [26] has maintained that the original scale with a general factor and six subscales still presents the closest alignment with the theoretical conceptualization of the construct, which is an important aspect of any psychometric measure [38]. She has also argued that empirical evidence supports the notion that self-compassionate behavior includes both an increase in compassionate self-responding and a decrease in uncompassionate self-responding, so measuring one without the other would not represent the complete picture [24]. Recent studies have found support for a global self-compassion factor plus six subscale factors, where exploratory structural equation modeling (ESEM) and bifactor ESEM have been used [29, 30, 39].

Therefore, Neff et al. [29] recommended that future studies compare confirmatory factor analysis (CFA) models with ESEM when testing the validity of the SCS. CFA models are too constrained for multidimensional measures, as they require subscale items to load onto a single target factor, with the non‐target loadings constrained to zero [40]. This often leads to incorrect assumptions about measurement error when indicators are loaded onto other factors [41]. Since the SCS is a multidimensional measure, ESEMs are appropriate because they account for the fact that factor indicators might reflect more than one thing [42]. Specifically, Neff et al. [29] argued that bifactor ESEM could more accurately model the SCS, as it assumes the presence of a global factor and specific subscale factors, allowing for the cross-loading of items that are constrained to be as close to zero as possible [40]. Furthermore, the bifactor ESEM provides the closest alignment to Neff’s [23] conceptualization of the self-compassion construct. In this study, the factor structure of the SCS was therefore examined through targeted CFA, bifactor CFA, ESEM, and bifactor ESEM analyses, in line with the procedures followed by Neff et al. [29] and Pommier et al. [43].

### Gender differences in the measurement of self-compassion

Measurement invariance is also an important aspect of scale validity, as it directly affects the interpretability of results [44]. Tests for measurement invariance compare responses to scale items across different groups (for example, gender or age) to ensure that no systematic differences exist in how the groups interpret the items. If systematic differences were to be found, a researcher would be unable to make inferences about any other relationships or differences [45]. Six different tests are commonly conducted, each looking at a different aspect of scale invariance. Configural invariance looks at whether factors load in the same way across groups; metric invariance looks for parity in the actual factor loadings across groups; scalar invariance examines the item thresholds across groups; strict invariance checks that the variance is the same across groups; latent variance‒covariance checks that the data vary in the same way and that the relationships between the latent variables are the same across groups; and finally, latent mean invariance examines whether the means of the latent variables are the same across the groups [46].

In the past, studies have been conducted to assess the measurement invariance of the SCS, but few have done so using the bifactor ESEM [39]. Given that Neff [26] insists that bifactor ESEM provides the best model fit, it makes sense that tests for measurement invariance can also be conducted using this model. Studies by Tóth-Király et al. [30] and Tóth-Király and Neff [39] found support for all measures of invariance, except for latent mean invariance. In this study, measurement invariance was tested across genders because there have been mixed results relating to gender differences in self-compassion; some studies have found that females have lower self-compassion levels than men [47–49], while other studies have found no gender differences [50]. Therefore, testing the measurement invariance of the bifactor ESEM of the SCS was included as a second study objective.

### Self-compassion and Flourishing of Managers

Flourishing is a holistic and multidimensional measure of well-being that is conceptualized as a sense of emotional, psychological, and social well-being [51–53]. A growing body of research has examined flourishing at work, since well-being and decent work are included as global sustainable development goals [54]. Flourishing at work means that individuals enjoy and function well in all aspects of their jobs [55]. Studies have shown that employees who flourish in their work benefit individuals and organizations. From an individual perspective, flourishing employees experience more job satisfaction, have a greater sense of meaning, have better workplace relationships, and feel more positive at work [56, 57]. From an organizational perspective, flourishers are also more committed, less likely to leave, more engaged, hard-working, and do not take as many sick days as employees who are not flourishing [58–60]. The flourishing of managers is deemed particularly important because of their effect on every aspect of organizational life, including the behavior of the employees reporting to them [61, 62]. If managers flourish, there will be a positive impact on themselves, those who report to them, and the organization.

Several scales have been developed to measure flourishing, many of which have been validated and used extensively in the literature, including Diener’s Flourishing Scale [63] and Keyes’ Mental Health Continuum [57]. Adapting the work of Keyes [57], Rothmann [55] developed a separate scale for examining flourishing in an organizational context, and proposed a model in which emotional, psychological, and social well-being were explicitly linked to the work context. Although flourishing at work seems to be a crucial factor for individual performance, team functioning and organizational thriving, there is still much debate about the most appropriate and useful personal resources for facilitating flourishing. Apart from its close links with all aspects of well-being, there are several other reasons why self-compassion may be seen as one such personal resource. First, it acts as a buffer against the effects of high job demands; second, it is an enduring positive self-evaluation and refers to a general compassionate attitude toward oneself; third, it has protective benefits in the same way as resilience and becomes more motivating over time; and finally, self-compassion has been shown to regulate an individual’s response to stress before a stressor happens, so that it alters a person’s perception of what is and is not stressful rather than just affecting their responses after a stressful event has occurred [64].

### Self-compassion and Flourishing Profiles

This study adopted a person-centered approach to the flourishing of managers and used latent profile analysis (LPA) to examine the associations between the constructs. Latent profile analysis allows for the extraction of heterogeneous profiles of flourishing based on the patterns of managers’ responses rather than forcing them into a single overarching dimension. Specifically, it identifies whether groups of people (profiles) respond to items on a scale similarly, but differently from other groups [65]. These profiles can be analyzed further to determine whether their associations with other variables differ, allowing for tailoring interventions and recommendations towards specific groups in a more useful and directed way [66]. Previous studies have found different flourishing profiles and linked these profiles to levels of social support structures, stress, job satisfaction and organizational citizenship behavior, showing significant differences between the profiles in relation to these aspects [67, 68]. Establishing flourishing profiles of managers and examining these profiles in relation to self-compassion were therefore set as objectives for this study.

### Current Study

Several gaps have been noted in the literature. First, scientific information regarding the validity, reliability, and measurement invariance of the SCS in non-WEIRD contexts is lacking [69]. Validating the scale in a South African managerial context would demonstrate that it can accurately measure self-compassion in organizations and across different cultures, which is important for future research. Latent profile analysis offers an individualized understanding of how self-compassion and flourishing interact within different subgroups of people, which will shed light on whether differences exist in how managers benefit from self-compassion. Finally, because of their significant influence, establishing the links between self-compassion and managers’ flourishing can guide practical interventions that improve the well-being and mental health of all people in organizations.

This study aimed to investigate the SCS’s psychometric properties (factorial validity, reliability measurement invariance across genders) and assess the associations between self-compassion and flourishing profiles within a sample of South African managers.

The following hypotheses were proposed based on the study objectives:

- Hypothesis 1: The SCS is a valid (Hypothesis 1a), invariant for males and females (Hypothesis 1b), and reliable (Hypothesis 1c) measure of self-compassion.
- Hypothesis 2: Managers’ self-compassion is positively associated with their flourishing profiles.

## Method

A cross-sectional research design was deemed appropriate to achieve this, and a quantitative approach allowed for measuring the constructs [29, 70].

### Participants

All students registered for postgraduate management qualifications at South African business schools were invited to participate, as these qualifications require previous work and management experience. Participation was voluntary, and informed consent was obtained online from each respondent before they completed the survey. A total of 390 completed responses were received, of which slightly more were from males (*N* = 207) than from females (*N* = 183). The mean age of the participants was 39.98 years (*SD* = 8.10). The mean number of years in a management role was 9.16, where 34.6% were new managers (less than five years in management), 25.1% were experienced (six to 10 years in management), and 33.3% were very experienced (more than 10 years in management).

### Measures

*The Self-Compassion Scale* (SCS; 23) was used to measure self-compassion. The scale has 26 items, with six underlying subscales of self-compassion: self-kindness (five items; “I try to be understanding and patient towards those aspects of my personality I don’t like”); self-judgment (five items; “I’m disapproving and judgmental about my own flaws and inadequacies”); common humanity (four items; “I try to see my failings as part of the human condition”); isolation (four items; “When I think about my inadequacies, it tends to make me feel more separate and cut off from the rest of the world”); mindfulness (four items; “When something painful happens, I try to take a balanced view of the situation”); and overidentification (four items; “When I’m feeling down, I tend to obsess and fixate on everything that’s wrong”). Items are rated on a scale varying from 1 (*almost never*) to 5 (*almost always*). The scores of the six subscales were added together to obtain a total self-compassion score after the negative items were reverse-coded. The scale has been widely used and has good construct and convergent validity and reliability, with Cronbach’s alpha scores greater than 0.80 [23, 29].

*The Flourishing-at-Work Scale – Short Form* (FAWS-SF; 70) was used to measure managers’ flourishing at work. The scale has 18 items, with three underlying subscales: emotional well-being (four items; for example, “During the past month at work, how often did you feel happy?”); psychological well-being (nine items; for example, “During the past month at work, how often did you feel confident in expressing your own ideas and opinions?”); and social well-being (five items; for example, “During the past month at work, how often did you feel that you truly belonged to your organization?”). Responses to the FAWS-SF items are scored on a scale, which ranges from 1 (*never*) to 6 (*every day*) and indicates the frequency of experienced symptoms of well-being. The scale has shown good validity and reliability in previous South African studies [70].

### Procedure

The Economic and Management Sciences Research Ethics Committee (EMS-REC) at North‒West University in South Africa approved the study (NWU-01309–21-A4). Thereafter, all 19 accredited business schools in South Africa were approached, and nine of these agreed to participate (seven public and two private business schools). Further ethics clearances and permissions were then granted at each participating school. All business school students registered for postgraduate management qualifications were invited to participate in the online survey.

### Data analysis

The analyses were conducted using IBM SPSS 27 for Windows [71] and Mplus 8.10 [72]. The full information likelihood method (FIML; [73]) was used to address missing values. The SCS was analyzed using the weighted least squares mean and variance estimator (WLSMV) because it is suitable for categorical variables with five or fewer answers [29], while the maximum likelihood estimator (MLR) was used for the analysis of the FAWS-SF and the LPA.

**Fig. 1:**
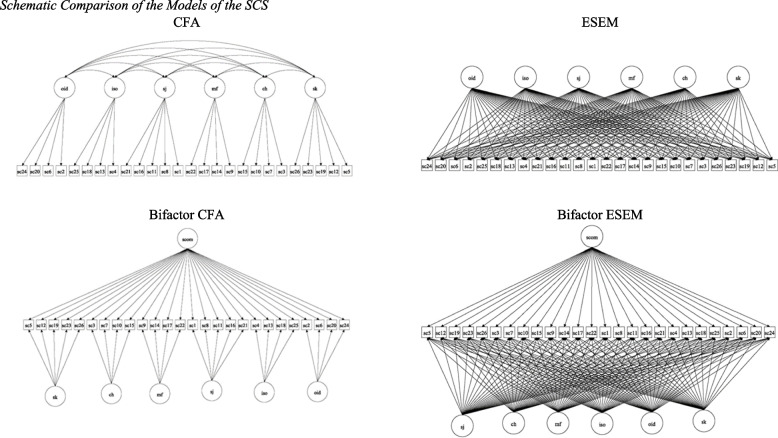
Schematic comparison of the models of the SCS. Note. CFA = confirmatory factor analysis; ESEM = exploratory structural equation modeling. Circles represent latent variables; squares represent scale items. One-headed full arrows represent factor loadings, and two-headed arrows represent factor correlations

Following the procedure suggested by Morin et al. [38] and Morin [42], the factor structure of the SCS was explored through targeted CFA, bifactor CFA, ESEM, and bifactor ESEM analyses (see the models in Fig. 1). Testing these four models corresponds with the procedure followed by Neff et al. [29]. The ESEM and bifactor ESEM were specified in Mplus 8.10 using a code generator [74]. Target rotation was applied, whereby a confirmatory approach to ESEM was taken. The fit of the four models was assessed using the comparative fit index (CFI), Tucker‒Lewis index (TLI), standardized root mean residual (SRMR), and root mean square error of approximation (RMSEA). The model fit criteria are as follows [42, 75, 76]: a nonsignificant (*p* > 0.05) chi-square (χ^2^) value; 0.90 and 0.95 (and higher) for CFI and TLI; 0.06 (and lower) for RMSEA; and 0.08 (and lower) for SRMR. According to West et al. [76], the CFI, TLI, RMSEA, and SRMR cut-off values can be used as rough guidelines for the model’s overall fit.

Tests for measurement invariance were conducted to assess the validity of the SCS for different genders. A code generator [77] was used to specify models to test the configural, metric, scalar, and strict invariance, as well as the latent variance‒covariance and latent mean invariance of the CS in Mplus 8.10. To compare the models for invariance, the criteria proposed by Chen [45] were applied, suggesting decreases in CFI and TLI ≤ 0.01 and increases in RMSEA ≤ 0.015 between a model and a more parsimonious one, which would support invariance [45]. These criteria have more recently been supported by Morin [42] and Preacher and Yaremych [78].

Kang et al. [79] noted that goodness of fit indices may vary depending on measurement quality; therefore, the following additional criteria were also used as indicators of measurement quality: factor loadings and item uniqueness > 0.10 but < 0.90 and explained common variance (ECV) and reliabilities [80]. The reliability of all scales used in the study was assessed using McDonald’s omega [81]. A bifactor indices calculator was used to calculate the reliability indices [82]. According to Hayes and Coutts [83], a value of 0.70 can be used as a cut-off value for scale reliability, while higher values are preferable. When assessing the reliabilities of bifactor CFA and ESEMs, it is important to remember that omega values will naturally be lower due to the variance that is shared between the general and specific factors. As a result, the accepted threshold of 0.70 is unrealistic, and 0.50 is suggested as a more realistic cut-off value [42, 84].

LPA in Mplus 8.10 [72] was used to identify the flourishing profiles of the managers. The number of potential profiles varied between one and five to find the best model, and different criteria were used to compare the models. Since there are no clear cut-off values for evaluating the optimal number of profiles, the Akaike information criterion (AIC), Bayesian information criterion (BIC), and sample-size adjusted Bayesian information criterion (aBIC) were compared, where lower values indicate a better fit [65]. The Vuong-Lo-Mendell-Rubin likelihood ratio test (VLMR-LRT), the adjusted Lo-Mendell-Rubin likelihood ratio (aLMR) test, and the bootstrapped likelihood ratio test (BLRT) were used to compare the current solution to the previous solution to select the right number of profiles. A nonsignificant p-value for a model with an additional profile meant that the previous model was supported [65]. Entropy values were used to examine the quality of the classification, where values between 0.60 and 1 indicated an acceptable classification [85]. The average latent class probabilities were used to assess the likelihood of the model accurately predicting the individuals in that profile (class membership), where a score close to 1 (but above 0.90) was a good indicator of member probability [72]. More recently, Weller et al. [86] suggested that values above 0.80 can be considered adequate if all the other information criteria are acceptable. Finally, the class size and interpretability were assessed to ensure that there were enough respondents in each profile and that the different profiles made sense [86].

In line with current best practices, LPA was conducted independently of the variables under study [87]. Bolck-Croon-Hagenaars (BCH) analysis was then used to compare the mean of the general self-compassion factor across each flourishing profile [88, 89]. BCH is the preferred approach since it is the most robust, yielding unbiased estimates under several conditions [90].

## Results

The empirical results are discussed in two parts: the validity, reliability, and measurement invariance of the SCS and the association of self-compassion with well-being .

### Factorial Validity, Reliability, and Invariance of the SCS

#### Factorial Validity

In line with the recommendations of Neff et al. [29], no higher-order models were tested, as these are not theoretically consistent and have not been supported in previous studies. As a result, the CFA, bifactor CFA, ESEM, and bifactor ESEMs were tested (Fig. 1) and the fit indices are presented in Table 1.

**Table 1:** Goodness-of-fit Indices for the SCS

Models	χ^2^	*df*	CFI	TLI	RMSEA	90% CI for RMSEA	SRMR
Six-factor CFA	780.22*	284	.95	.95	.07**	[.06,.07]	.04
ESEM	439.41*	184	.98	.96	.06**	[.05,.07]	.02
Bifactor CFA	1523.81*	273	.88	.86	.11*	[.10,.11]	.07
Bifactor ESEM	340.80*	164	.98	.97	.05	[.05,.06]	.02

*CFA* confirmatory factor analysis, *ESEM* exploratory structural equation modeling, *χ*^*2*^ weighted least squares chi-square test of exact fit, *df* degrees of freedom, *CFI* comparative fit index, *TLI* Tucker‒Lewis index, *RMSEA* root mean square error of approximation, *90% CI* 90% confidence interval of the RMSEA, *SRMR* standardized root mean square residual. AIC, BIC and aBIC values are not reported because of the estimator used

^*^ *p* <.05; ** *p* <.01

The six-factor CFA model showed satisfactory fit indices (both CFI and TLI = 0.95; RMSEA = 0.07 [0.06, 0.07, *p* = 0.001]; and SRMR = 0.04); however, as with previous studies, there was significant cross-loading between items, where some items showed high factor loadings (above 0.50) on the target subscale as well as several other nontarget subscales. As shown in recent studies [28–30], the higher-order factor model (bifactor CFA) was not supported, with both the CFI and TLI below 0.90. In contrast, the ESEM and bifactor ESEM both showed excellent fit statistics, but the bifactor ESEM outperformed all the other models, with CFI and TLI values of 0.98 and 0.97, respectively, SRMR of 0.02, and RMSEA of 0.05 [90% CI 0.05, 0.06]. Table 2 shows the factor loadings for the different models.

**Table 2:** Standardized parameter estimates for the four measurement models of the SCS

Variable		CFA	ESEM						Bifactor CFA		Bifactor ESEM						
IC	Item	SF (λ)	SK (λ)	CH (λ)	M (λ)	SJ (λ)	I (λ)	OI (λ)	GF (λ)^a^	SF (λ)	GF (λ)^a^	SK (λ)	CH (λ)	M (λ)	SJ (λ)	I (λ)	OI (λ)
SK	SCS5	.74^**^	.65^**^	.18^**^	.28^**^	-.25^**^	-.13^**^	-.02	-.62^**^	.44^**^	-.58^**^	.40^**^	.18^**^	.23^**^	-.21^**^	.04	.16^**^
SK	SCS12	.82^**^	.66^**^	.20^**^	.28^**^	-.24^**^	-.10^**^	-.23^**^	-.71**	.39^**^	-.69^**^	.34^**^	.14^**^	.16^**^	-.07^*^	.16^**^	.08^*^
SK	SCS19	.81^**^	.69^**^	.13^**^	.30^**^	-.28^**^	-.16^**^	−10^**^	-.69**	.42^**^	-.66^**^	.41^**^	.10^**^	.19^**^	-.14^**^	.04	.12^*^
SK	SCS23	.67^**^	.49^**^	.34^**^	.19^**^	-.11^**^	-.19^**^	-.17^**^	-.58**	.33^**^	-.58^**^	.39^**^	.21^**^	-.09	.30^**^	.06	-.13^*^
SK	SCS26	.80^**^	.57^**^	.31^**^	.23^**^	-.19^**^	-.30^**^	-.12^**^	-.69**	.40^**^	-.66^**^	.43^**^	.18^**^	.01	.11	-.07	-.06
CH	SCS3	.53^**^	.11^*^	.41^**^	.26^**^	-.27^**^	-.19^**^	.17^**^	-.36**	.31^**^	-.29^**^	.12^**^	.42^**^	.12^*^	-.11^**^	-.23^**^	-.15^**^
CH	SCS7	.60^**^	.32^**^	.64^**^	.15^**^	-.05	.09^*^	.04	-.32**	.72^**^	-.28^**^	.15^**^	.67^**^	.10^**^	-.02	.14^**^	.11^*^
CH	SCS10	.76^**^	.39^**^	.67^**^	.16^**^	.03	.00	-.22^**^	-.48**	.60^**^	-.47^**^	.10^*^	.58^**^	.07^*^	.16^**^	.22^**^	.12^**^
CH	SCS15	.77^**^	.37^**^	.46^**^	.31^**^	-.14^**^	-.12^**^	.04	-.52**	.40^**^	-.43^**^	.25^**^	.44^**^	.18^**^	.01	-.03	-.03
M	SCS9	.68^**^	.26^**^	.11^**^	.60^**^	-.03	-.17^**^	-.28^**^	-.55**	.43^**^	-.55^**^	.03	.03	.48^**^	.15^**^	.04	-.10^*^
M	SCS14	.75^**^	.28^**^	.21^**^	.69^**^	-.06	-.25^**^	-.14^**^	-.60**	.61^**^	-.56^**^	.07^*^	.18^**^	.55^**^	.08^*^	-.10^**^	-.08^*^
M	SCS17	.83^**^	.45^**^	.24^**^	.57^**^	-.03	-.25^**^	-.20^**^	-.69**	.45^**^	-.65^**^	.18^**^	.17^**^	.44^**^	.13^**^	-.02	.01
M	SCS22	.76^**^	.54^**^	.30^**^	.35^**^	−13^**^	-.06	-.19^**^	-.65**	.21^**^	-.59^**^	.29^**^	.24^**^	.22^**^	.05	.16^**^	**.01**
SJ	SCS1	.62^**^	-.40^**^	.02	.04	.39^**^	.19^**^	.30^**^	.55^**^	.28^**^	.58^**^	-.15^**^	.13^**^	.20^**^	.18^**^	-.07	-.02
SJ	SCS8	.69^**^	-.23^**^	-.10^**^	.09^*^	.52^**^	.27^**^	.40^**^	.60^**^	.43^**^	.65^**^	.13^**^	.01	.25^**^	.33^**^	-.03	-.01
SJ	SCS11	.68^**^	-.22^**^	-.03^**^	-.07	.59^**^	.31^**^	.19^**^	.59^**^	.44^**^	.59^**^	-.04	.04	.13^**^	.34^**^	.14^**^	.14^**^
SJ	SCS16	.77^**^	-.25^**^	-.15^**^	-.07^*^	.43^**^	.43^**^	.32^**^	.69^**^	.25^**^	.71^**^	.05	-.02	.13^**^	.14^**^	.15^**^	.04
SJ	SCS21	.82^**^	-.29^**^	-.10^**^	-.20^**^	.57^**^	.31^**^	.30^**^	.74^**^	.30^**^	.73^**^	-.02	-.01	.02	.30^**^	.07	.13^**^
ISO	SCS4	.80^**^	-.35^**^	.01	-.13^**^	.22^**^	.48^**^	.49^**^	.75^**^	.14^**^	.78^**^	.11^**^	.16^**^	-.02	.02	**.08**	-.19^**^
ISO	SCS13	.81^**^	-.13^**^	-.06^*^	-.23^**^	.35^**^	.76^**^	.18^**^	.70^**^	.55^**^	.71^**^	.08^**^	.07^*^	-.00	.04	.52^**^	.06
ISO	SCS18	.78^**^	-.18^**^	.01	-.21^**^	.35^**^	.71^**^	.16^**^	.67^**^	.54^**^	.68^**^	.04	.11^**^	-.02	.08^*^	.50^**^	.02
ISO	SCS25	.83^**^	-.29^**^	-.08^*^	-.12^**^	.32^**^	.46^**^	.49^**^	.77^**^	.17^**^	.79^**^	.11^*^	.09^**^	.06	.05	**.08**	-.04
OID	SCS2	.77^**^	-.33^**^	.08	-.09^*^	.29^**^	.45^**^	.47^**^	.73^**^	.10^*^	.74^**^	.01	.26^**^	.11^**^	-.01	.07	**-.02**
OID	SCS6	.80^**^	-.25^**^	-.03	-.08^*^	.28^**^	.41^**^	.63^**^	.75^**^	.13^**^	.80^**^	.20^**^	.17^**^	.10^*^	-.01	-.03	**-.03**
OID	SCS20	.79^**^	-.13^**^	-.07	-.37^**^	.49^**^	.29^**^	.44^**^	.72^**^	.48^**^	.74^**^	.07	.10^**^	-.10	.07	.03	.40^**^
OID	SCS24	.77^**^	-.05	-.05	-.44^**^	.52^**^	.26^**^	.42^**^	.69^**^	.44^**^	.70^**^	.13^**^	.09^*^	-.14^**^	.15^**^	.04	.40^**^

*CFA* confirmatory factor analysis, *ESEM* exploratory structural equation modeling, *IC* item component, *SF* loading on the respective specific factor when cross-loadings were constrained to zero, *SK* self-kindness, *CH* common humanity, *M* mindfulness, *SJ* self-judgment, *I* isolation, *OID* overidentification, *SCS* self-compassion scale, *GF* general factor; λ = standardized factor loadings. The target loadings are in bold. ^a^ The self-compassion factor (GF) was reverse-scored in the Mplus analyses; therefore, negative correlations denote a positive relationship, and vice versa

^*^p <.05 **p <.01

Factor loadings for the CFA model were generally high. As with previous studies, however, the CFA results were problematic because factor correlations between the three positive factors (*r* = 0.75 to 0.86, mean = 0.80) and the three negative factors (*r* = 0.88 to 0.94, mean = 0.91) were high, while the intercorrelations between the positive and negative factors were moderate (*r* = 0.34 to 0.71, mean = 0.56). The factor loadings for the ESEM were slightly lower than those for the CFA solution, however, the specific dimensions were better differentiated, with fewer problematic cross-loading items (as previous studies have also shown).

In line with the study objectives and as Neff et al. [29] recommended, the bifactor ESEM was particularly interesting because it fits best with the underlying theory. Therefore, these factor loadings were examined in greater detail. Here, the factor loadings of the general factor showed how much of the global variance each item explained, while the loadings for each subscale indicated the amount of unique variance explained by that subscale over and above the general factor [91]. A detailed look at how the items loaded onto the general factor showed moderate to high factor loadings across the board, except for two of the four items on the common humanity subscale. The four common humanity items loaded higher onto their specific factor than any other subscale factors, which shows that this subscale explained more unique variance than the other subscale factors. In addition, items from the three negative subscales showed higher loadings on the general factor than items from the three positive subscales.

When examining the loadings on the specific factors, the three positive subscales were well-defined and showed greater specificity than the negative subscales. The negative subscales were less well-defined. Several items on the negative subscales showed high loadings on the general factor but very low loadings on their specific factor. Notably, two items on the OID subscale (items SC2 and SC6) showed high loadings, respectively, on the general factor but very low loadings on their own factor, implying that they might be better indicators of general self-compassion than of their own dimension. The overall results of the validity tests indicated that the SCS was a valid measure of self-compassion, and Hypothesis 1a was therefore supported.

#### Reliability of the SCS

The bifactor ESEM of the SCS was used as a basis for the omega estimates provided, and Table 3 includes an in-depth analysis of the scale’s reliability following the guidelines provided by Rodriguez et al. [81].

**Table 3:** Reliability indices of the SCS (Bifactor ESEM)

	**ECV (S&E)**	**ECV (NEW)**	**Omega/OmegaS**	**Relative Omega**	**H**	**FD**
General SC factor	.73	.73	.96	.95	.95	.97
Self-kindness	.05	.28	.86	.28	.48	.76
Common humanity	.08	.67	.74	.67	.64	.82
Mindfulness	.05	.36	.82	.34	.51	.78
Self-judgement	.03	.14	.83	.13	.28	.61
Isolation	.04	.19	.89	.14	.41	.78
Overidentification	.02	.13	.87	.06	.27	.67

*ECV* explained common variance (where ECV-S provides the additional variance explained by all the items that load onto a specific factor), *ECV NEW* explained common variance (where ECV-S provides the additional variance explained by only the items that are meant to load on that specific factor), *Omega/OmegaS* the internal reliability of the scale (where OmegaS is the relative strength of a specific factor), *OmegaH/OmegaHS* Omega Hierarchical, which indicates the percentage of variance in item scores that is accounted for by the general factor, *Relative Omega* OmegaH divided by Omega, which indicates the overall variance accounted for by the general factor (for specific factors, this indicates the percentage of variance in the subscale that is independent of the general factor), *H* a measure of the replicability of a construct; *FD* factor determinacy [81]

The bifactor ESEM showed good reliability for the general factor and for each subscale, with omega values ranging from 0.74 (common humanity) to 0.96 (general SC factor), which is well above the acceptable cut-off of 0.70 [81]. The omega hierarchical value of 0.91 for the general self-compassion factor showed that this accounted for more than 90% of the reliable variance in item response, where values > 0.80 indicate good reliability of the total score [92]. The omega hierarchical value for the subscales (OmegaHS) showed the additional unique variance explained by the items on each subfactor after accounting for the variance that had been attributed to the general factor [92]. Once again, the positive subscales were more well defined than the negative subscales, accounting for more variance over and above the general factor. Specifically, the relative omega value for the common humanity subscale was 0.67, which was almost double that of any of the other subscales and indicated a very well-defined construct. Nonetheless, all the subscales explained sufficient additional variance to support their inclusion in the model. These results indicated that Hypothesis 1c was supported, as the SCS is a reliable measure of self-compassion.

#### Measurement invariance of the SCS

Table 4 shows the measurement invariance scores of the SCS across genders, including configural, metric, scalar, and strict invariance, as well as latent mean invariance and latent variance‒covariance invariance.

**Table 4:** Measurement invariance of the SCS – Bifactor ESEM Model

Model	χ^2^ (*df*)	*p*	CFI	TLI	RMSEA	90% CI	SRMR	CM	Δχ^2^	Δ*df*	ΔCFI	ΔTLI	ΔRMSEA	ΔSRMR
Configural invariance	509.24 (328)	.001**	.98	.97	.05	[.04,.06]	.02	-	-	-	-	-	-	-
Metric invariance	666.44 (461)	.001**	.98	.97	.05	[.04,.06]	.03	1	216.21*	133	-.00	.01	-.01	.01
Scalar invariance	726.13 (532)	.001**	.98	.98	.04	[.04,.05]	.03	2	92.71	71	.00	.01	-.01	.00
Strict invariance	792.48 (558)	.001**	.98	.97	.05	[.04,.05]	.04	3	45.86*	26	-.00	-.00	.00	.00
Latent variance‒covariance invariance	66.40 (586)	.018*	.99	.99	.03	[.01,.04]	.05	4	36.71	28	.02	.02	-.02	.01
Latent mean invariance	71.91 (593)	.001*	.99	.99	.03	[.02,.04]	.05	5	26.94*	7	-.00	-.00	.01	.00

*CFI* comparative fit index, *TLI* Tucker‒Lewis index, *RMSEA* root mean square error of approximation, *90% CI* 90% confidence interval of the RMSEA, *SRMR* standardized root mean square residual; Δχ^2^ chi-square difference test calculated using the Mplus DIFFTEST option reported for descriptive purposes, *CM* comparison model

^*^ *p* <.05; ** *p* <.01

The results showed three significant changes in chi-square values, which might indicate a lack of measurement invariance; however, chi-square values are often sensitive to sample size, and alternative fit indices (AFIs) must, therefore, always be examined [45]. The AFIs showed acceptable CFI and TLI values for all tests (between 0.97 and 0.99) as well as acceptable RMSEA and SRMR values ≤ 0.05. In addition, the criteria for changes in CFI and TLI (0.01 or less) and changes in RMSEA and SRMR (less than 0.01 and 0.03) were upheld in all the tests, except the test for latent variance‒covariance, indicating that the variance‒covariance between and within the latent variables was not the same for males and females [45]. Tóth-Király et al. [30] and Tóth-Király and Neff [39] showed similar results where measurement invariance was supported across all the tests, except for the latent mean variance. These results indicated that the SCS was invariant across gender, and Hypothesis 1b was supported.

### Latent profile analysis and associations between self-compassion and flourishing

The results of the LPA are presented below.

#### Validity and reliability of the FAWS-SF

To prepare the FAWS-SF data for latent profile analyses, the validity of the three-factor structure of the FAWS-SF was tested. Table 5 shows the fit statistics of the different CFA models tested, while Table 6 depicts the subscale factor loadings and the omega values. CFA was conducted using the MLR, as used in previous studies [55]. The model fit statistics (Model 1), including those of two model revisions (Models 1a and 1b), are presented in Table 5 below.

**Table 5:** Fit Statistics of the FAWS-SF CFA

Models for three-factor CFA	AIC	BIC	aBIC	χ^2^	*df*	CFI	TLI	RMSEA [90% CI for RMSEA]	SRMR
Model 1	20,784.46	21,010.53	20,829.67	544.57*	132	.89	.87	.09** [.08,.10]	.06
Model 1a (Items 12 and 13 correlated)	20,630.80	20,860.84	20,676.81	422.51*	131	.92	.91	.08** [.07,.08]	.05
Model 1b (Items 21 and 22 correlated)	20,599.77	20,833.78	20,646.57	396.73*	130	.93	.91	.07** [.06,.08]	.05

*CFI* comparative fit index, *TLI* Tucker‒Lewis index, *RMSEA* root mean square error of approximation, *90% CI* 90% confidence interval of the RMSEA, *SRMR* standardized root mean square residual

^*^ *p* <.05; ** *p* <.01

**Table 6:** Factor Loadings and Reliability for the FAWS-SF CFA

Component	Factor loading range	Mean	*SD*	McDonald’s omega
Emotional well-being	.63 to.84	.75	.09	.83
Psychological well-being	.62 to.85	.71	.09	.91
Social well-being	.65 to.89	.78	.10	.90

The fit indices for Model 1 showed that the three-factor structure was not supported, as the CFI and TLI values were below the cut-off of 0.90, and the RMSEA was above the cut-off of 0.08 for acceptable fit. The modification index (MI) values indicated that the model fit could be improved if Item 12 (“During the past month at work, how often did you feel that your work makes a difference to the world?”) and Item 13 (“During the past month at work, how often did you feel that the work you do serves a greater purpose?”) were allowed to correlate (MI = 113.64). Both items are on the psychological well-being subscale, and both relate to finding meaning in work, so it was not unexpected that they would be highly correlated. Several authors have argued that it is unrealistic to expect no correlations when working with multidimensional constructs and that it is better to revise the models while incorporating the correlated errors [40, 93]. The revised Model 1a showed a great improvement, with lower AIC and BIC values; CFI = 0.92, TLI = 0.91, RMSEA = 0.08, and SRMR = 0.05, all of which showed acceptable fit. However, a look at the MI values of the revised Model 1a indicated that there could be further improvement in the model fit if Items 20 (“During the past month at work, how often did you feel that people in your organization are basically good?”) and 21 (“During the past month at work, how often did you feel that the way your organization works makes sense to you?”) were also allowed to correlate (MI = 26.85). The results of this further modification (Model 1b) showed improvements in the AIC and BIC values, which were lower, as well as improvements in the CFI (0.93) and RMSEA (0.07) values, indicating strong support for the validity of the scale.

The factor loadings for all items in the FAWS-SF were moderate to high. The omega values indicated that the FAWS-SF was a reliable and valid measure.

### Latent profiles of flourishing

LPA was conducted to establish the correct number of flourishing profiles for managers. The fit indices for each model are shown in Table 7.

**Table 7:** Comparison of the Fit Indices for the Different Latent Profile Models

Model	AIC	BIC	aBIC	VLMR LR *p* value	aLMR LR *p* value	BLRT *p* value	Entropy	Smallest class (%)
1-profile	3673.21	3697.01	3677.97					
2-profile	3156.12	3195.78	3164.05	.001*	.001*	.001*	.83	47.95
3-profile	2935.19	2990.72	2946.30	.001*	.001*	.001*	.85	21.28
4-profile	2890.03	2961.42	2904.31	.177	.187	.001*	.80	21.03
5-profile	2863.93	2951.18	2881.38	.098	.104	.001*	.80	3.85

*Note*. *AIC* Akaike information criterion, *BIC* Bayesian information criterion, *ABIC* Adjusted Bayesian information criterion, *LMR LR* Lo-Mendell-Rubin likelihood ratio test, *ALMR LR* Adjusted Lo-Mendell-Rubin likelihood ratio test, *BLRT* Bootstrap likelihood ratio test

^*^p <.01

When choosing the correct model, all relevant criteria were assessed. In this case, the two-class LPA was easily rejected, as the three-class LPA improved the AIC, BIC, and aBIC. Similarly, the five-class LPA model was easily rejected because the size of Class 2 was very small (*N* = 15), which could cause problems with further statistical analysis and could affect the interpretability of findings [94]. The three-class model met all the specific information criteria, with AIC, BIC, and aBIC values lower than those of the two-class model; significant VLMR, aLMR, and BLRT *p*-values; an acceptable entropy value (0.85); and average latent class probabilities all above 0.90. However, the four-class profile was finally adopted as the true population model for several reasons. First, the AIC, BIC, and aBIC values were lower than those of the three-class model, which are very important criteria [94]. Second, although the VLMR and aLMR values were not significantly different, the BLRT value was significant (*p* < 0.001), which has proven to be the most accurate and consistent of the three likelihood ratio tests [94]. Finally, in terms of interpretability, the four profiles made more sense and provided richer information when differentiated that way [95], and recent literature has identified similar profiles [68].

**Fig. 2:**
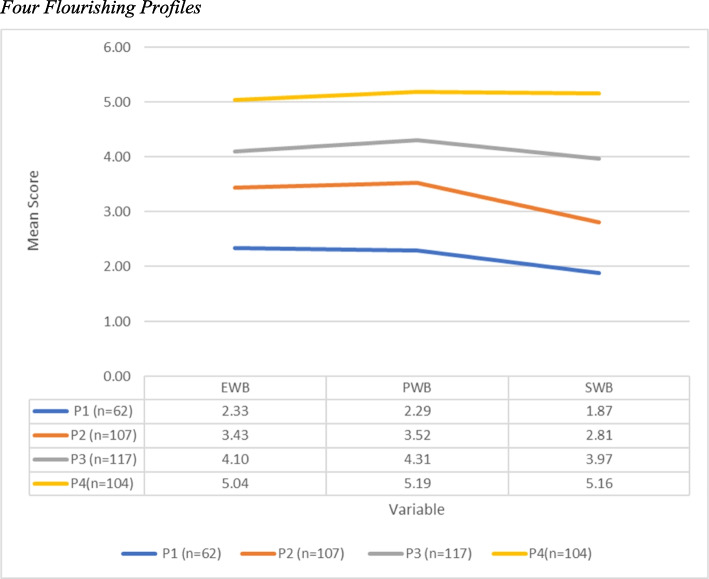
Four flourishing profiles

The profiles depicted in Fig. 2 can be interpreted as follows: (a) Profile 1: languishers (16%) – managers in this profile scored low on all three flourishing subscales, but especially on the social well-being subscale. (b) Profile 2: moderate languishers (27.4%) – managers in this group scored moderately low on all three flourishing subscales, especially on the social well-being subscale. (c) Profile 3: moderate flourishers (30%) – managers in this group scored moderately high on all three flourishing subscales, although the social well-being subscale score was lower than the other subscale scores. (d) Profile 4: flourishers (26.6%) – managers in this group scored high on all three flourishing-at-work subscales, and the social well-being score was similar to, or higher than, the other subscale scores.

### Associations between self-compassion and latent flourishing profiles

Using the BCH method, the mean levels of self-compassion were then estimated across each profile using the general self-compassion factor and the six latent factors. The BCH method uses a weighted multiple-group analysis to identify which groups correspond to which latent profile and is a robust method for use with both categorical and continuous distal outcome variables [90]. Table 8 shows the results of the equality tests, but only those subscales where significant relationships were found are included.

**Table 8:** Equality Tests of Means Across Profiles Using the BCH Procedure with Three Degrees of Freedom for the Overall Test

Variable	General self-compassion		Self-judgement		Isolation		Overidentification		Self-kindness	
	Mean	SE	Mean	SE	Mean	SE	Mean	SE	Mean	SE
1. Languishing	-.38	.13	0.48	.10	-.38	.11	-.34	.10	.22	.10
2. Moderately languishing	-.20	.10	0.08	.08	.02	.08	-.04	.08	.12	.08
3. Mod flour	-.13	.11	−0.14	.08	-.12	.09	-.04	.09	-.14	.07
4. Flour	.55	.10	−0.22	.07	.33	.09	.28	.08	-.09	.08
Chi-square tests	χ^2^	*p*	χ^2^	*p*	χ^2^	*p*	χ^2^	*p*	χ^2^	*p*
Overall test	44.17	.001**	43.00	.001**	28.94	.001**	25.05	.001**	12.34	.006**
Profile 1 vs 2	1.11	.292	9.25	.002**	7.72	.005**	4.64	.031*	0.62	.432
Profile 1 vs 3	2.36	.124	26.13	.001**	3.63	.057	5.31	.021*	8.20	.004**
Profile 1 vs 4	32.25	.001**	35.06	.001**	27.35	.001**	23.46	.001**	5.52	.019*
Profile 2 vs 3	0.18	.668	3.31	.069	1.20	.273	0.00	.995	4.64	.031*
Profile 2 vs 4	27.95	.001**	8.65	.003**	6.95	.008**	8.18	.004**	3.36	.067
Profile 3 vs 4	18.51	.001**	0.58	.446	12.35	.001**	6.59	.010*	.18	.671

*Note.* Profile 1 = languishers; Profile 2 = moderate languishers; Profile 3 = moderate flourishers; Profile 4 = flourishers

^*^ *p* <.05; ** *p* <.01

The results in Table 8 established a close relationship between self-compassion and flourishing and supported Hypothesis 2. In terms of the general self-compassion factor (GSC), the results of the BCH indicated that the four profiles differed in terms of their general self-compassion means. Profile 1 (languishers) showed the lowest levels of self-compassion (−0.38), while Profile 4 (flourishers) showed high levels of GSC (0.55). Overall, significant differences existed between the GSC levels of the different flourishing profiles. Specifically, the results showed that statistically significant differences existed for GSCs between Profiles 1 and 4, Profiles 2 and 4, and Profiles 3 and 4. In other words, the flourishers (Profile 4) were significantly different from all the other profiles in terms of their levels of self-compassion.

Further tests for each subscale showed that all three negative subscales (self-judgment, isolation, and overidentification) also showed significant differences between the profiles (*p* < 0.001) and that levels of uncompassionate self-responding were highest for the languishers and lowest for the flourishers. Interestingly, the results of the negative subscales almost mirrored those of the general self-compassion scale, where Profile 4 (flourishers) was found to be significantly different from the other profiles, whereas the positive subscales showed very different results. Self-kindness was the only positive subscale to show overall significant differences between profiles (*p* < 0.006), where Profile 1 (languishers) was found to be significantly different from Profiles 3 and 4 (moderate flourishers and flourishers) in terms of their levels of self-kindness. The overall mindfulness and common humanity subscales tests showed no significant differences between the profiles.

## Discussion

These results support Neff’s argument [26] that the SCS is a psychometrically valid and reliable measure of self-compassion and that the bifactor ESEM provides the best fit, both statistically and theoretically, with a general factor and six well-defined subscales. In particular, the validity and reliability scores for the general self-compassion factor strongly support the use of this scale as a measure of global self-compassion, which is in line with several recent studies [24, 30, 39]. The items loading onto the general factor showed moderate to high factor loadings across the board. Authors who have argued that the SCS should be separated into two scales for compassionate and uncompassionate self-responding [35, 36] have not compared CFA with ESEM models and have not considered how this separation would undermine the theoretical consistency of the scale [26]. The validation of the scale also shows that it is suitable for use in non-WEIRD and organizational contexts. In their 2021 study, Tóth-Király and Neff [39] found support for the SCS as a valid and reliable scale across many different samples and cultures, so these findings extend those claims. This potentially opens new avenues for research on the links between self-compassion and effective leadership in South African organizations, which is a key area for future study.

Our results confirmed the significant direct relationship between self-compassion and flourishing of managers, supporting Hypothesis 2. This finding is important in the context of the organizational flourishing literature. How managers behave at work and how they relate to their colleagues has a significant impact on all employees and on every aspect of organizational life and functioning [62, 96, 97]. Promoting behaviors that improve flourishing should therefore be a priority for all organizational directors and heads. Interventions focused on increasing self-compassion in individuals have shown remarkable results, with significant increases in compassionate self-responding and significant decreases in uncompassionate self-responding in a short time [22]. Therefore, self-compassion should be included in all management development programs, helping managers relate to themselves in kinder and more caring ways and to be less judgmental of themselves and their flaws. Furthermore, organizations and leaders should have more support structures and greater intentions to cultivate and practice self-compassion, perhaps including self-compassionate behaviors as part of annual performance reviews.

The LPA provided valuable insights into the relationship between the constructs, and showed four distinct flourishing profiles for managers: flourishers, moderate flourishers, moderate languishers, and languishers. Levels of uncompassionate self-responding (measured by three negative subscales) were significantly higher in all other profiles (languishers, moderate languishers, and moderate flourishers) than they were in the flourishing profile, indicating a strong negative relationship between uncompassionate self-responding and flourishing, and once again supporting the idea that interventions aimed at reducing managers’ self-criticism could have a meaningful impact on their capacity to flourish. Interestingly, the same was not true for the positive subscales, where only self-kindness showed significant differences between the four flourishing profiles, while common humanity and mindfulness did not. Many studies have found similar differences between positive and negative self-responding and their relationships to well-being [31, 33] and Neff [26] argues that this is to be expected. In the high-pressure environments in which managers operate, flourishing may require more than just being kind to oneself. Managers might be self-compassionate but still face external stressors that limit their ability to flourish.

Finally, the individualized nature of the LPA analysis showed that the flourishing managers’ profiles differed significantly from the other profiles in terms of social well-being. This was the only profile in which the social well-being scores were the same as or higher than the other subscale scores (emotional and psychological well-being). It is possible that managers with greater self-compassion also have better social networks and relationships with colleagues at work, which makes a difference to their overall flourishing. Maintaining positive functional relationships with colleagues and subordinates can be the difference between success and failure as a manager, and it makes sense that this might be a differentiating factor in their flourishing. To foster good working relationships, organizations should promote networking and teambuilding and create opportunities for managers and employees to connect on a personal level to create authentic and respectful relationships. Performance management and reward systems could also be aligned with these behaviors, to ensure that values of trust and respect are upheld.

The study had several limitations which should be acknowledged. Firstly, the cross-sectional design does not provide insight into how these constructs or relationships might change over time. Future organizational studies should include self-compassion interventions and use an appropriate methodology, such as a randomized controlled trials to examine this aspect. Other intervention studies would also be especially useful in examining the organizational conditions under which self-compassion might best be cultivated to improve the long-term flourishing of managers and employees. Secondly, self-selection bias might mean that the participants were already interested in aspects of personal development and well-being, and that this sample does not accurately represent all managers in South Africa [98]. Future research could use a more randomized sampling method to avoid this. Finally, further exploring how other variables like organizational culture, leadership expectations, and external pressures affect the relationship between self-compassion and flourishing could provide valuable insights.

## Conclusion

This study strongly supports Neff’s [26] argument to retain the SCS in its current form, with a general factor and six subfactors, a finding that has significant implications for future research. A strong and significant relationship was found between self-compassion and the flourishing of managers. Using LPA, four profiles of flourishing managers were identified, which were shown to be significantly different in terms of their levels of general self-compassion. Therefore, managers should intentionally cultivate and practice self-compassion, it should be included in management training programs, and supported by performance and reward systems to improve managers’ flourishing at work and benefit their colleagues, subordinates, and organizations more broadly.

## Data Availability

The datasets generated and analysed for this study can be found in Mendeley Data, V1, doi: 10.17632/8ydb6ct53n.1

## Abbreviations

AFI: Alternative fit indices
BIC: Bayesian information criterion (BIC)
BCH: Bolck-Croon-Hagenaars
BLRT: Bootstrapped likelihood ratio test
CFI: Comparative fit index
CFA: Confirmatory factor analysis
EMS-REC: Economic and Management Sciences Research Ethics Committee
ECV: Explained common variance
ESEM: Exploratory structural equation modelling
FAWS-SF: Flourishing-at-Work Scale – Short Form
FIMS: Full information likelihood method
GSC: General self-compassion factor
LPA: Latent profile analysis
MLR: Maximum likelihood estimator
MI: Modification index
NWU: North‒West University
RMSEA: Root mean square error of approximation
SCS: Self-Compassion Scale
SRMR: Standardized root mean residual
TLI: Tucker‒Lewis index
VLMR-LRT: Vuong-Lo-Mendell-Rubin likelihood ratio test
WLSMV: Weighted least squares mean and variance estimator
WEIRD: Western, Educated, Industrialized, Rich, Democratic
